# Logical definition-based identification of potential missing concepts in SNOMED CT

**DOI:** 10.1186/s12911-023-02183-7

**Published:** 2023-05-09

**Authors:** Xubing Hao, Rashmie Abeysinghe, Kirk Roberts, Licong Cui

**Affiliations:** 1 School of Biomedical Informatics, University of Texas Health Science Center at Houston, Houston, TX USA; 2 Department of Neurology, McGovern Medical School, University of Texas Health Science Center at Houston, Houston, TX USA

**Keywords:** Ontology and terminology, Ontology enrichment, Logical definition, Summarization model, Concept mapping

## Abstract

**Background:**

Biomedical ontologies are representations of biomedical knowledge that provide terms with precisely defined meanings. They play a vital role in facilitating biomedical research in a cross-disciplinary manner. Quality issues of biomedical ontologies will hinder their effective usage. One such quality issue is missing concepts. In this study, we introduce a logical definition-based approach to identify potential missing concepts in SNOMED CT. A unique contribution of our approach is that it is capable of obtaining both logical definitions and fully specified names for potential missing concepts.

**Method:**

The logical definitions of unrelated pairs of fully defined concepts in non-lattice subgraphs that indicate quality issues are intersected to generate the logical definitions of potential missing concepts. A text summarization model (called PEGASUS) is fine-tuned to predict the fully specified names of the potential missing concepts from their generated logical definitions. Furthermore, the identified potential missing concepts are validated using external resources including the Unified Medical Language System (UMLS), biomedical literature in PubMed, and a newer version of SNOMED CT.

**Results:**

From the March 2021 US Edition of SNOMED CT, we obtained a total of 30,313 unique logical definitions for potential missing concepts through the intersecting process. We fine-tuned a PEGASUS summarization model with 289,169 training instances and tested it on 36,146 instances. The model achieved 72.83 of ROUGE-1, 51.06 of ROUGE-2, and 71.76 of ROUGE-L on the test dataset. The model correctly predicted 11,549 out of 36,146 fully specified names in the test dataset. Applying the fine-tuned model on the 30,313 unique logical definitions, 23,031 total potential missing concepts were identified. Out of these, a total of 2,312 (10.04%) were automatically validated by either of the three resources.

**Conclusions:**

The results showed that our logical definition-based approach for identification of potential missing concepts in SNOMED CT is encouraging. Nevertheless, there is still room for improving the performance of naming concepts based on logical definitions.

## Background

An ontology is a knowledge representation artifact, containing a taxonomy as proper part. Representations of an ontology are intended to designate certain combinations of classes with precisely defined meanings, and relations between them [1]. Ontology terms (also called classes or concepts) are typically common nouns or noun phrases [2]. Ontology terms are linked together through relations such as “*is-a (subtype)*” or “*part-of*” that form the graph structure of the ontology [2]. Biomedical ontologies provide solutions to manage, curate, and analyze huge volumes of digital unstructured textual content generated in biomedical research and practice. They play a vital role in knowledge management; data integration, exchange and semantic interoperability; and decision support and reasoning [3].

Although ontology curators seek ways to ensure that the ontologies are as accurate and comprehensive as possible, quality issues inevitably exist. Besides, knowledge in the biomedical domain is constantly growing and accordingly biomedical ontologies are evolving. Thus, quality assurance (QA) of biomedical ontologies in various aspects needs to be performed such as maintaining accuracy, consistency, completeness, and soundness [4]. However, this can be complicated and infeasible to perform manually for large ontologies with complex structures. Therefore, automated or semi-automated tools that uncover potential quality issues are critical to relieve the burden of manual review.

In this work, we focus on the quality issue of missing concepts (related to completeness) in SNOMED CT. We first develop an approach that leverages logical definitions of hierarchically unrelated concept pairs in non-lattice subgraphs to come up with logical definitions of potential missing concepts, where a hierarchically unrelated concept pair refers to two concepts without an IS-A relationship. Then, we fine-tune a text summarization model (called PEGASUS) to predict the fully specified names of potential missing concepts from the logical definitions. In addition, the potential missing concepts we identified are validated in three ways: the Unified Medical Language System (UMLS), biomedical literature in PubMed, and a newer version of SNOMED CT. Overall, we identified 23,031 potential missing concepts in the March 2021 US Edition of SNOMED CT of which 2,312 (10.04%) were validated. The result indicated that our approach is encouraging to uncover potential missing concepts in SNOMED CT as well as being generalizable to many other biomedical ontologies. To the best of our knowledge, our work is the first that attempts to identify potential missing concepts that are equipped with both fully specified names and logical definitions.

### SNOMED CT

SNOMED CT provides a common language that supports communication between different specialties and sites of care. It plays an important role in indexing, storing, retrieving, and aggregating clinical data [5]. Specifically, the SNOMED CT United States (US) Edition is the official source of SNOMED CT for use in US healthcare systems, combining the content of both the US Extension and the International releases of SNOMED CT [6]. In this work, we use the March 2021 release of the US Edition of SNOMED CT [7].

**Fig. 1:**
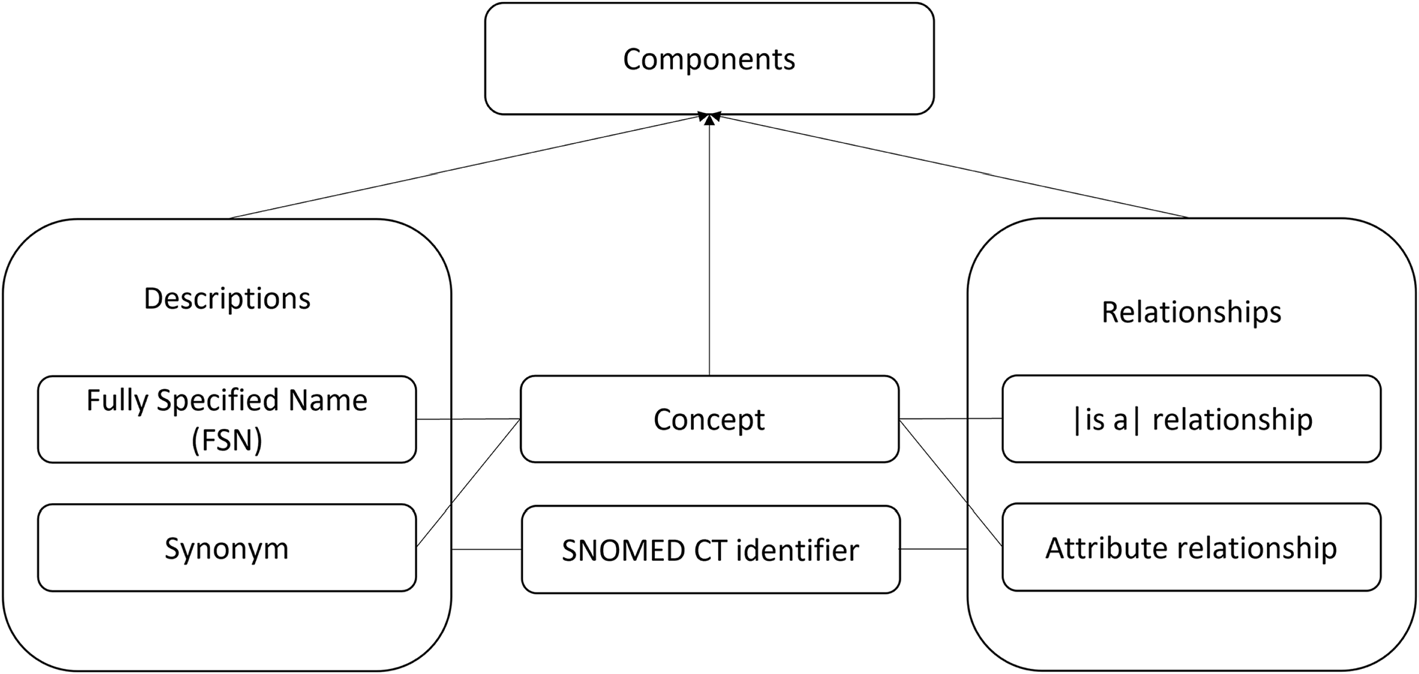
SNOMED CT logical model. This figure is adopted and reproduced referencing [8]

The core structure of SNOMED CT is provided by its logical model. It outlines how the components can be managed in an implementation setting to meet a variety of primary and secondary uses, representing the terminology’s essential content [8]. The components of the logical model shown in Fig. 1 include the identifier, concept descriptions, and relationships. Each SNOMED CT component has a unique integer identifier [9]. Concept descriptions include the fully specified name (FSN) and the synonyms. The FSN is a term that names the meaning of a concept in a manner that is intended to be unambiguous and stable across multiple contexts [10]. A synonym is an acceptable way to express the meaning of a concept in a certain language or dialect [11]. A description is marked as the “preferred term” if it is considered the most clinically suitable way to express a concept in a clinical record [12]. Relationships reflect associations between concepts and are used to logically define the meaning of a concept in such a way that can be processed computationally. Relationships include *is-a* relationship and attribute relationship [8]. Each concept other than the root concept has at least one *is-a* relationship and can have as many attribute relationships as needed, which form the logical definition of the concept.

### Non-lattice subgraphs

**Fig. 2:**
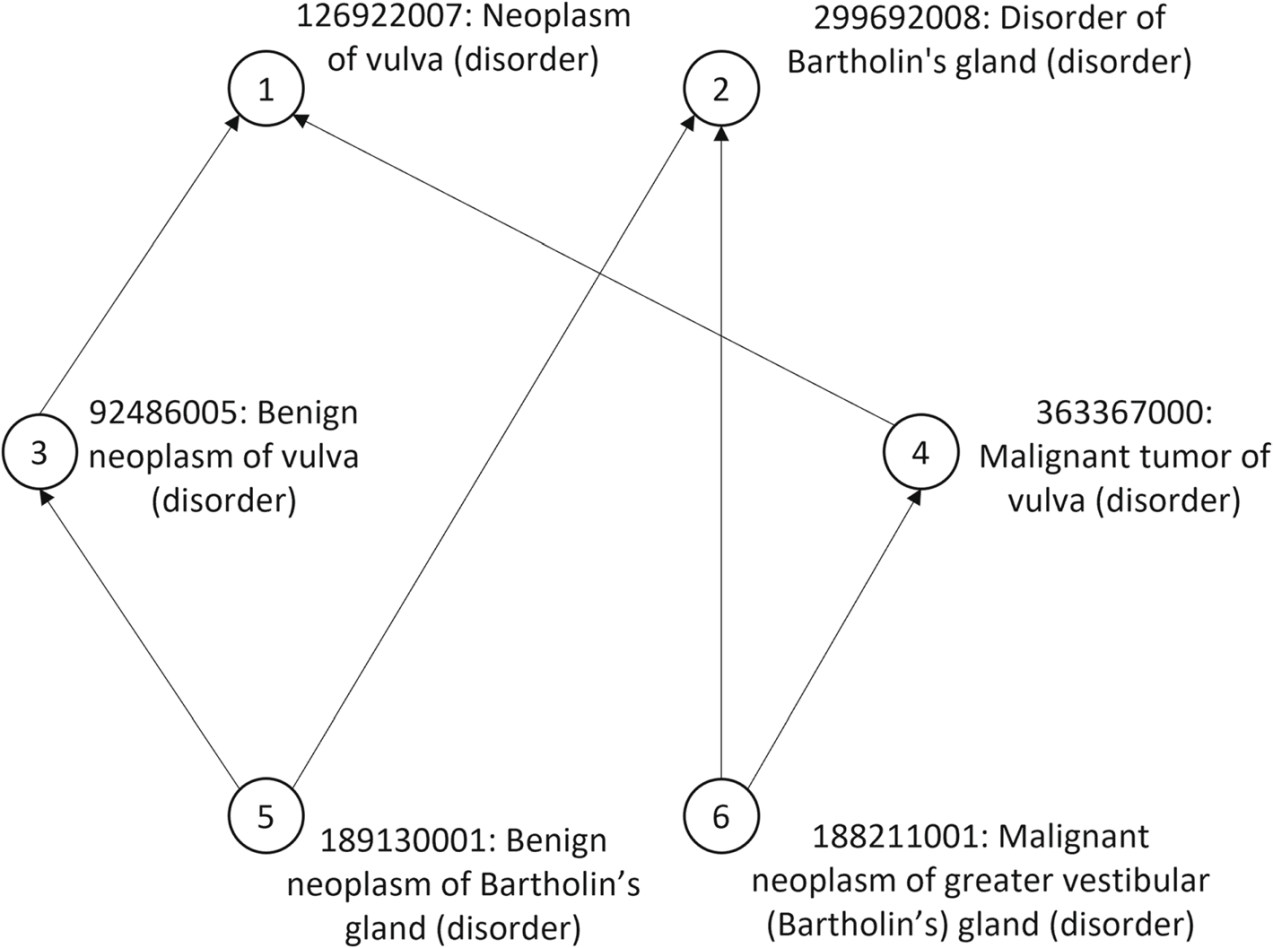
A non-lattice subgraph of size 6 in SNOMED CT. This non-lattice subgraph is generated from the non-lattice concept pair: (“*Neoplasm of vulva (disorder)*”, “*Disorder of Bartholin’s gland (disorder)*”)

Being a lattice is considered to be a desirable property of the hierarchical structure of an ontology [13]. In a lattice, any two nodes have a unique maximal shared descendant and a unique minimal shared ancestor [14]. A pair of concepts that share more than one maximal common descendant or minimal common ancestor is called a non-lattice pair. Analyzing non-lattice pairs themselves may entail redundant analysis as multiple non-lattice pairs may have the same maximal common descendants. Therefore, to avoid redundancies and simplify analysis, non-lattice subgraphs have been introduced [15]. Non-lattice subgraphs are often indicative of ontology defects such as missing concepts or missing hierarchical relations. There have been various methods developed to compute non-lattice pairs in an ontology [13, 16–18]. A non-lattice subgraph can be generated by reversely computing the minimal common ancestors of the maximal common descendants of a non-lattice pair and aggregating all concepts and *is-a* relations between them [17]. Figure 2 shows an example of a non-lattice subgraph in the March 2021 release of the SNOMED CT (US Edition) generated from the non-lattice pair: (“*Neoplasm of vulva (disorder)*”, “*Disorder of Bartholin’s gland (disorder)*”). The concepts in this non-lattice pair share two maximal common descendants “*Benign neoplasm of Bartholin’s gland (disorder)*” and “*Malignant neoplasm of greater vestibular (Bartholin’s) gland (disorder)*”. Between the non-lattice pair and the maximal common descendants, there exist two concepts “*Benign neoplasm of vulva (disorder)*” and “*Malignant tumor of vulva (disorder)*”. These concepts and the *is-a* relations between them form the non-lattice subgraph. Note that the size of a non-lattice subgraph is defined as the number of concepts it contains [14]. Therefore, the above mentioned non-lattice subgraph in Fig. 2 is of size 6.

### Summarization models

Summarization models aim at generating accurate, concise, and linguistically fluent summaries that cover the principal information in the input document. While extractive summaries contain only words in the input, abstractive summaries may include novel words and re-phrasings [19]. This aspect of abstractive summarization is important for proposing concept names from logical definitions (and why we have chosen to use such a model here) since the names of many intermediate concepts do not include words from the names of their related concepts. Recent studies such as MASS (Masked Sequence to Sequence pre-training) [20], BART (denoising sequence-to-sequence pre-training) [21], and T5 (Text-to-Text Transfer Transformer) [22] leverage pre-trained transformer-based sequence-to-sequence models. They have achieved success in language generation-based tasks including abstractive summarization. In 2020, Zhang et al. proposed PEGASUS, a sequence-to-sequence abstractive summarization model trained with gap-sentence generation as a pre-training objective [19]. Unlike above-mentioned MASS, BART, and T5 that mask words or smaller continuous text spans, PEGASUS masks whole sentences from documents and studies strategies for selecting these sentences. The architecture of PEGASUS is a standard transformer (with both a 16-layer encoder and a 16-layer decoder stack). Beyond this, there are two noteworthy aspects of PEGASUS that makes it a strong abstractive summarization model: it has a specialized pre-training algorithm to better learn summaries, and it is pre-trained on a very substantial amount of data. The new pre-training objective in PEGASUS is gap-sentence generation. During the pre-training process, important sentences (defined as those with the highest n-gram overlap with the rest of the document) are masked from the original input document. Then these gap-sentences are concatenated into a pseudo-summary [19]. They used two large text corpora C4 (Colossal and Cleaned version of Common Crawl) [22] and HugeNews for pretraining, and validated their PEGASUS model on 12 downstream datasets including XSum [23], CNN/Daily Mail [24], NEWSROOM [25], Multi-News [26], Gigaword [27], arXiv and PubMed [28], BIGPATENT [29], WikiHow [30], Reddit TIFU [31], AESLC [32], and BillSum [33]. PEGASUS achieved state-of-the-art performance on all 12 downstream datasets.

### Related work on missing concept identification

A number of ontology quality assurance approaches have been investigated to identify missing concepts in biomedical ontologies. In general, these can be categorized into two types: (1) importing concepts from another knowledge source; and (2) exploiting the knowledge within the terminology [34].

For example, He et al. have proposed vertical topological patterns to import missing concepts from external ontologies to SNOMED CT and National Cancer Institute thesaurus (NCIt) [35–37]. They have investigated instances where different intermediate concepts exist between mapped concepts across two ontologies. UMLS has been leveraged to map concepts across ontologies. Such topological patterns indicated the possibility to import concepts from one ontology to another [35–37]. Chandler et al. proposed a similarity-based approach to recommend concepts from a text corpus to SNOMED CT [38]. The approach involved extraction of candidates from the text corpus that are represented with certain features. Then important features were identified and used to recommend missing concepts to SNOMED CT. In [39] and [40], lexico-syntactic patterns have been explored to enrich different biomedical ontologies including NCIt from unstructured clinical text.

On the other hand, there have been studies leveraging the knowledge within the terminology to identify missing concepts. For instance, in [15], a specific lexical pattern called “Union-Intersection” in non-lattice subgraphs was introduced to suggest potential missing concepts in SNOMED CT. In [34] and [41], Zheng et al. have investigated formal concept analysis (FCA)-based approaches leveraging the lexical features of concept names to identify potential missing concepts in NCIt and SNOMED CT. Then the UMLS and biomedical literature in PubMed [42] were leveraged to automatically validate the missing concepts identified. More recently, we explored a lexical-based intersection approach based on non-lattice subgraphs to identify potential missing concepts in SNOMED CT [43]. We conducted an order-preserving intersection of lexical features of unrelated concept pairs in non-lattice subgraphs to suggest potential missing concepts, which were further validated by utilizing the external knowledge in the UMLS and PubMed.

Regardless of identifying missing concepts based on external or internal knowledge, the above-mentioned existing works were not capable of providing logical definitions for missing concepts.

## Methods

Our logical definition-based approach has five main steps. We first obtain the derived logical definition of each concept. Second, we pre-compute all non-lattice subgraphs of SNOMED CT and extract candidate concept pairs. Next, we intersect the derived logical definitions of the candidate concept pairs to generate the logical definitions of the potential missing concepts. Then we fine-tune a PEGASUS text summarization model and use it to predict the fully specified names of potential missing concepts based on the generated logical definitions. Finally, the identified potential missing concepts are validated by using external resources including the UMLS, biomedical literature in PubMed, and the newer September 2022 US Edition of SNOMED CT.

### Derived logical definition generation

SNOMED CT releases both stated and inferred logical definitions for all concepts. We use inferred logical definitions of concepts in this work.

A concept’s own logical definition consists of one or more attribute groups. Each attribute group consists of one or more attribute-value pairs. The attributes are grouped to avoid ambiguity as to how they apply [44]. In this work, we consider each *is-a* relation to be in a separate attribute group in addition to the existing attribute groups of the concept. For example, Table 1 shows the attribute groups in the logical definition of concept “*Malignant epithelial neoplasm of thyroid (disorder)*”. Note that its *is-a* parent “*Malignant tumor of thyroid gland (disorder)*” is in a separate group in addition to the already existing two attribute groups.

**Table 1:** The three attribute groups in the logical definition of concept “*Malignant epithelial neoplasm of thyroid (disorder)*”

Group Number	Logical Definition Group
0	{(*Is a, “Malignant tumor of thyroid gland (disorder)*”)}
1	{(*Is a, “Malignant epithelial neoplasm (disorder)*”)}
2	{(*Associated morphology (attribute), “Malignant epithelial neoplasm - category (morphologic abnormality)”), (Finding site (attribute), “Thyroid structure (body structure)”)}*

A concept’s derived logical definition is obtained by aggregating attribute groups from two sources:

1. the attribute groups in the logical definition of the concept itself; and
2. the attribute groups that are more general than those obtained above.

Given an attribute group $G_1$ with attribute-value pairs $\{(r_{(1,1)}, v_{(1,1)}),(r_{(1,2)}, v_{(1,2)}),\ldots , (r_{(1,m)}, v_{(1,m)})\}$ and another attribute group $G_2$ with attribute-value pairs $\{(r_{(2,1)}, v_{(2,1)}), (r_{(2,2)}, v_{(2,2)}), \ldots ,(r_{(2,n)}, v_{(2,n)})\}$ where $G_1\ne G_2$. If for each attribute-value pair $(r_{(2,j)}, v_{(2,j)})$ in $G_2$, there exists an attribute-value pair $(r_{(1,i)}, v_{(1,i)})$ in $G_1$ such that $(r_{(2,j)}, v_{(2,j)})$ is the same as or more general than $(r_{(1,i)}, v_{(1,i)})$, then $G_2$ is considered as more general than $G_1$. Here an attribute-value pair $(r_2, v_2)$ is considered to be more general than another attribute-value pair $(r_1, v_1)$ if one of the following conditions is met:

1. $r_1 = r_2$ and $v_1$ *is-a* $v_2$; or $r_1$ *is-a* $r_2$ and $v_1 = v_2$; or $r_1$ *is-a* $r_2$ and $v_1$ *is-a* $v_2$. For instance, consider the attribute-value pairs (*is-a, “Chelating agent adverse reaction (disorder)*”) and (*is-a, “Edetate adverse reaction (disorder)*”). They have the same attribute *is-a* (i.e., $r_1 = r_2$). Since “*Edetate adverse reaction (disorder)*” is a subtype of “*Chelating agent adverse reaction (disorder)*”, we consider (*is-a, “Chelating agent adverse reaction (disorder)*”) to be more general than (*is-a, “Edetate adverse reaction (disorder)*”).
2. $v_1$ contains an attribute-value pair $(r_b, v_2)$ in its logical definition such that $r_a \circ r_b$ is a subproperty of $r_2$ , where $r_1=r_a$ or $r_1$ *is-a* $r_a$, and $\circ$ denotes a property chain. Here, a property chain is a rule used to infer the existence of a property from a chain of properties [45]. For example, consider the attribute-value pairs (*Causative agent (attribute), “Sodium calcium edetate (substance)*”) and (*Causative agent (attribute), “Edetate (substance)*”). The concept “*Sodium calcium edetate (substance)*” has an attribute-value pair (*Is modification of (attribute), “Edetate (substance)*”). Since SNOMED CT has the property chain *Causative agent* $\circ$ *Is modification of* is a subproperty of *Causative agent*, we say that the attribute-value pair (*Causative agent (attribute), “Edetate (substance)*”) is more general than the attribute-value pair (*Causative agent (attribute), “Sodium calcium edetate (substance)*”).

**Fig. 3:**
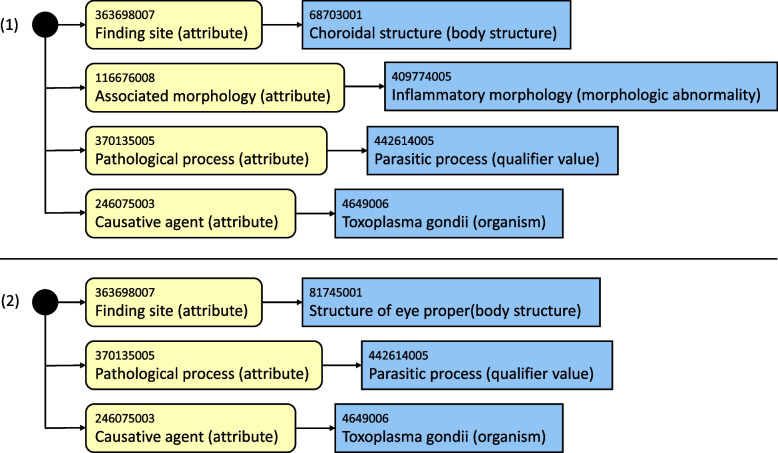
Two example attribute groups. Attribute group (2) is more general than attribute group (1)

Figure 3 shows an example where one attribute group is more general than another. Here, both the attribute groups (1) and (2) have the attribute-value pairs (*Pathological process (attribute), “Parasitic process (qualifier value)*”) and (*Causative agent (attribute), “Toxoplasma gondii (organism)*”) in common. However, the attribute-value pair (*Finding site (attribute), “Structure of eye proper (body structure)*”) in the attribute group (2) is more general than the attribute-value pair (*Finding site (attribute), “Choroidal structure (body structure)*”) in the attribute group (1). This is because of the relation: “*Choroidal structure (body structure)*” *is-a* “*Structure of eye proper (body structure)*”.

### Candidate concept pair generation

As mentioned earlier, non-lattice subgraphs are often indicative of various quality issues of biomedical ontologies. Therefore, we focus on concept pairs in non-lattice subgraphs to identify missing concepts in this work. First we extract all non-lattice subgraphs in the March 2021 release of the US Edition of SNOMED CT using an efficient non-lattice detection algorithm [18]. For simplicity of analysis, we focus on the non-lattice subgraphs with size less than 10.

A pair of concepts (*A*, *B*) is considered to be a candidate pair if it satisfies the following conditions:

- both *A* and *B* are within the same non-lattice subgraph;
- *A* and *B* do not have any direct or indirect *is-a* relation between them;
- both *A* and *B* are fully defined concepts (i.e., concepts with one or more sufficient definitions that can distinguish itself and its subtypes from other concepts) [46]; and
- the level of concept *A* $\ge 10$ and the level of concept *B* $\ge 10$ (the level of a concept is the number of hops in the longest path from the root to the concept [47]), ensuring *A* and *B* are not very general concepts.

For instance, in the non-lattice subgraph shown in Fig. 2, the concepts “*Neoplasm of vulva (disorder)*” and “*Disorder of Bartholin’s gland (disorder)*” satisfy all the above conditions and form a candidate pair. Note that different non-lattice subgraphs may have some overlapping concepts and hence generate the same candidate pair. We remove such duplicated candidate pairs for further steps.

### Missing concept identification

Given a candidate concept pair, we obtain the logical definition of a potential missing concept by intersecting their derived logical definitions. In other words, the common attribute groups in the derived logical definitions of the candidate pair form the logical definition of a potential missing concept.

The logical definition of the potential missing concept obtained is further reduced as follows. Let the logical definition of the potential missing concept contain attribute groups {$G_1, G_2, ..., G_k$}. For an attribute group $G_m$ in this logical definition, if there exists another attribute group $G_n$ in the same logical definition where $G_m$ is more general than $G_n$, then $G_m$ will be removed from the logical definition.

If the logical definition obtained this way already exists as the logical definition of an existing concept in SNOMED CT, this will be ignored. We also ignore duplicated cases where intersecting different candidate pairs yield the same logical definition.

### Missing concept naming

Each potential missing concept obtained above only contains a logical definition. They should be aptly named before being considered to be included in SNOMED CT. In this work, we explore whether deep learning could be leveraged to predict the concept’s FSN from the logical definition. We frame this problem as a text summarization task. The aim is to summarize the logical definitions of the missing concepts to come up with their FSNs. To achieve this, we fine-tune a PEGASUS text summarization model.

#### Data preparation

We use all existing concepts in SNOMED CT and their logical definitions for our fine-tuning experiments. The March 2021 release of the US Edition of SNOMED CT contains 361,461 concepts in total. We split these concepts into three datasets as follows: training set with 289,169 concepts (80%), validation set with 36,146 concepts (10%), and testing set with 36,146 concepts (10%).

The PEGAUS model is provided with the logical definitions and FSNs of the concepts. The model is fine-tuned to summarize logical definitions to FSNs. The logical definitions need to be converted to text before feeding them to the model. We converted each logical definition of a concept to a sentence where each attribute-value-pair was separated by a comma. For instance, the concept “*Product containing digoxin (medicinal product)*” has two attribute-value pairs in its logical definition:

- {(*Is a, “Product containing glycoside (product)”*)} and
- {(*Has active ingredient*, “*Digoxin (substance)*”)}

These are converted to a sentence as follows: “*Is a Product containing glycoside (product), Has active ingredient Digoxin (substance).*”

#### Fine-tuning PEGASUS

We fine-tune the PEGASUS model up to 50 epochs and pick the checkpoint with the best validation performance. Table 2 summarizes the parameters used for fine-tuning. We use the AdamW optimizer [48] with an initial learning rate of 5e-5 which was linearly decreased during the fine-tuning process.

**Table 2:** The hyperparameters of fine-tuned PEGASUS

Parameter	Value
Learning rate	5e-5
Batch size per GPU	32
Maximum epochs	50
Optimizer	AdamW
Max input tokens	1024
Max target tokens	128
Metric for best model	Loss

Our evaluation metric is Recall-Oriented Understudy for Gisting Evaluation (ROUGE), which is widely used for evaluating automated summarization in natural language processing. ROUGE compares an automatically produced summary against a reference summary [49]. ROUGE-N is an n-gram recall between a candidate summary and a set of reference summaries. ROUGE-N is computed as follows [49]:

1 $$\begin{aligned} ROUGE-N=\frac{\sum _{S\in {\{ReferenceSummaries\}}}\sum _{gram_{n}\in {S}} Count_{match}(gram_{n})}{\sum _{S\in {\{ReferenceSummaries\}}}\sum _{gram_{n}\in {S}} Count(gram_{n})} \end{aligned}$$

where *ReferenceSummaries* are the summaries we know are correct (i.e., the actual FSNs of concepts in the testing set), $Count_{match}(gram_{n})$ is the number of matching n-grams that occur in both the reference and the proposed summary (i.e., the FSN summarized by the model), and $Count(gram_{n})$ is the number of n-grams in the reference summaries.

In this work, we calculate ROUGE-1 (unigram based scoring), ROUGE-2 (bigram based scoring), and ROUGE-L (longest common subsequence based scoring) [50].

### Missing concept name validation

We validate the predicted names of the potential missing concepts by leveraging three sources: (1) external terminologies in the UMLS; (2) biomedical literature in PubMed; and (3) a newer release of SNOMED CT.

#### Normalization

The FSNs of the potential missing concepts may not appear in the same form in the three sources mentioned above. Some words may be in their singular/plural forms or may be different but synonymous. Therefore, to handle such situations and ensure effective coverage when the FSNs are matched with other FSNs/other ontology concept names/biomedical text, normalization is performed as follows. First we delete extra white spaces, convert the name to lower case, and conduct lemmatization. We use the open-source python library Natural Language Toolkit (NLTK) for this step [51]. Then, if the name includes words corresponding to other concepts in SNOMED CT, we replace such words with the preferred synonym term of the corresponding SNOMED CT concept. For example, the word “dyspepsia” appears as a synonym of the SNOMED CT concept “*Indigestion (finding)*” of which “Indigestion” is the preferred term. So, we replace the word “dyspepsia” with “indigestion”. Finally, the stop words are removed from the name.

#### UMLS-based validation

The UMLS integrates many biomedical terminologies including SNOMED CT, Gene Ontology (GO), Medical Subject Headings (MeSH), and Human Phenotype Ontology (HPO) [52]. It contains over 16 million concept names from 218 source vocabularies, which are aggregated through more than 4 million UMLS concepts [53]. The basic building blocks of the UMLS are called atoms, which are concept names from different source vocabularies. Every UMLS atom is assigned an Atom Unique Identifier (AUI). The atoms from different vocabularies having the same meaning (i.e. they are synonymous) are grouped together to form a UMLS concept with a Concept Unique Identifier (CUI) [54]. For example, the UMLS concept “*Kidney Diseases*” (with CUI C0022658) has corresponding atom “*Kidney Disease*” (with AUI A0427003) from MeSH and atom “*Disease of kidney*” from SNOMED CT (with AUI A3399122). In this work, we leverage the concepts that are in English in the 2022-AA-full version of the UMLS.

In the UMLS-based validation, we similarly normalize all the UMLS atoms and check whether a match can be obtained between a normalized FSN of a potential missing concept and a normalized UMLS atom without considering the semantic tag of the FSN. If this is found, then we consider the potential missing concept to represent a valid case.

#### PubMed-based validation

PubMed contains about 34 million citations and abstracts of biomedical literature [55]. We use the 2022 baseline release of PubMed and its daily update files up to July 8th, 2022. If the FSN of a potential missing concept (not considering the semantic tag) appears as a base noun phrase in the title or abstract of a publication in PubMed, then it is considered to be a valid case. The requirement of base noun phrase is to make sure that the potential missing concept does not appear as a substring of another concept. For instance, a potential missing concept “*thoracic artery*” may exists in an abstract as a substring of “*fetal thoracic artery*”. These cases are ignored as in such instances the abstracts are not discussing particularly about the potentially missing concept we want to validate.

The title and abstract for each publication are extracted from the PubMed release files, and parsed with spaCy to identify base noun phrases [56]. Each base noun phrase is then normalized similar to FSN normalization discussed earlier. As the search space is very large, a sequential search for potential missing concepts among these base noun phrases would be time consuming. Therefore, we index the normalized noun phrases using the open-source search library Apache Lucene [57]. Then, we search the index for the normalized FSNs of potential missing concepts (not considering the semantic tag), which is significantly faster than directly performing a sequential search on the base noun phrases.

#### Validation based on a newer release of SNOMED CT

We leverage the newer September 2022 US Edition of SNOMED CT to check whether any of our identified potential missing concepts exists in this newer release. To reiterate, the potential missing concepts are obtained from the March 2021 US Edition of SNOMED CT. If the normalized FSN of a potential missing concept matches with a normalized FSN of a concept in the September 2022 release, or if the logical definition of the potential missing concept is the same as the logical definition of a concept in the September 2022 release, then the potential missing concept is considered to be a valid case.

## Results

### Candidate concept pair generation

There exists a total of 361,461 concepts in the March 2021 release of the US Edition of SNOMED CT. We obtained the derived logical definitions for all the concepts. There also exist 236,291 non-lattice subgraphs in this version of SNOMED CT in total. Out of these, 43,923 are with size $\le 10$, which contained 92,099 unique candidate pairs.

### Missing concept identification

Intersecting the derived logical definitions of 92,099 candidate pairs resulted in a total of 30,313 unique potential missing concepts with logical definitions.

### Missing concept naming

**Fig. 4:**
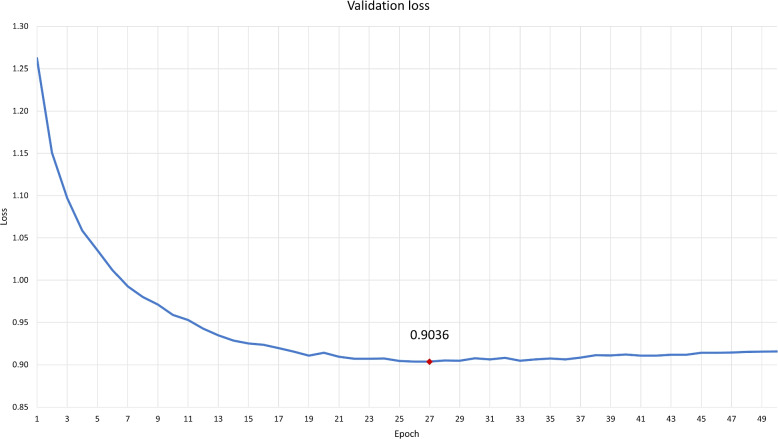
Validation loss throughout the fine-tuning process. The best model was found to be at epoch 27

We performed our fine-tuning experiments on a Linux server running CentOS 7.9.2009 with 8 NVIDIA A100 GPUs. Fine-tuning took 8 hours 2 minutes and 46 seconds in total. Figure 4 shows a plot of the validation loss at each epoch. The best model was found to be at the 27th epoch. The best model achieved ROUGE-1 of 72.83, ROUGE-2 of 51.06, and ROUGE-L of 71.76 on the test dataset.

We also compared the model’s prediction of the FSNs of the concepts in the test set with their actual FSNs after normalizing both. It was seen that the model correctly predicted 11,549 out of 36,146 concepts from the test dataset. Table 3 includes 5 examples of correct predictions by the model. In addition, there were 15 predicted FSNs of which only the semantic tag was different.

**Table 3:** Five correctly predicted concept FSNs in the test set by the PEGASUS mode

Logical definition	Correctly predicted FSN
{(*Is a, “Acute otitis media (disorder)*”)}	Acute myringitis (disorder)
{(*Is a, “Myringitis (disorder)*”)}	
{(*Finding site (attribute), “Tympanic membrane structure (body structure)”*),	
(*Associated morphology (attribute), “Acute inflammation (morphologic abnormality)*”)}	
{(*Clinical course (attribute), “Sudden onset AND/OR short duration (qualifier value)*”)}	
{(*Is a, “Chest pain (finding)*”)}	Dull chest pain (finding)
{(*Is a, “Dull pain (finding)*”)}	
{(*Finding site (attribute), “Thoracic structure (body structure)*”)}	
{(*Is a, “Ergot alkaloid measurement (procedure)*”)}	Lysergic acid diethylamide measurement (procedure)
{(*Component (attribute), “Lysergic acid diethylamide (substance)*”)}	
{(*Method (attribute), “Measurement - action (qualifier value)*”)}	
{(*Is a, “Medicinal product categorized by structure (product)*”)}	Product containing carbamazepine (medicinal product)
{(*Has active ingredient (attribute), “Carbamazepine (substance)*”)}	
{(*Is a, “Foreign body in bronchus (disorder)*”)}	Foreign body in main bronchus (disorder)
{(*Finding site (attribute), “Main bronchus structure (body structure)”*),	
(*Associated morphology (attribute), “Foreign body (morphologic abnormality)*”)}	

To obtain the FSNs of the 30,313 potential missing concepts, we used the best model found earlier with the logical definitions of these potential missing concepts. The model predicted 27,289 unique FSNs. Note that there were certain cases where the model predicted the same FSN for multiple potential missing concepts. We further checked if these FSNs already exist as FSNs for other concepts in the same SNOMED CT release. It was seen that the 4,089 FSNs matched with FSNs of existing concepts after normalization. In addition, 169 FSNs were observed to be matched with FSNs of concepts where only the semantic tag was different after normalization.

The remaining 23,031 concepts were considered as not already existing in SNOMED CT (hence potentially missing) and further validated by external resources.

### Missing concept validation

The UMLS-based validation revealed 1,227 out of 23,031 potential missing concepts existed in external terminologies in the UMLS. Table 4 demonstrates five examples of missing concepts validated by the UMLS. For instance, the missing concept with the FSN “*Cutaneous infection (disorder)*” was mapped to the UMLS atom “*Cutaneous infections*” with the AUI A24682086, which is a Human Phenotype Ontology (HPO) concept grouped under the UMLS CUI C1853193.

**Table 4:** Five examples of potential missing concepts validated by the UMLS

Missing concept FSN	Missing concept logical definitions	Mapped UMLS atom name/AUI/CUI
Primary cerebellar neoplasm (disorder)	{(*Is a, “Neoplasm of cerebrum (disorder)*”)}	Primary Cerebellar Neoplasm/A1639672(MSH)/C0750998
	{(*Finding site (attribute), “Structure of cerebrum (body structure)”*),	
	(*Associated morphology (attribute), “Neoplasm (morphologic abnormality)*”)}	
Cutaneous infection (disorder)	{(*Is a, “Infection of skin (disorder)*”)}	Cutaneous infections/A24682086(HPO)/C1853193
	{(*Finding site (attribute), “Skin structure (body structure)”*),	
	(*Associated morphology (attribute)*,	
	“*Morphologically abnormal structure (morphologic abnormality)*”)}	
	{(*Finding site (attribute), “Skin structure (body structure)”*),	
	(*Pathological process (attribute), “Infectious process (qualifier value)*”)}	
Congenital anomaly of lacrimal apparatus (disorder)	{(*Is a, “Congenital anomaly of lacrimal system (disorder)*”)}	congenital anomalies of lacrimal apparatus/A21020760(MEDCIN)/C3509790
	{(*Finding site (attribute), “Structure of lacrimal apparatus (body structure)”*),	
	(*Associated morphology (attribute)*,	
	“*Morphologically abnormal structure (morphologic abnormality)”*),	
	(*Occurrence (attribute), “Congenital (qualifier value)”*),	
	(*Pathological process (attribute)*,	
	“*Pathological developmental process (qualifier value)*”)}	
Lesion of foot (disorder)	{(*Is a, “Disorder of foot (disorder)*”)}	lesions feet/A13728295(MEDCIN)/C0744147
	{(*Finding site (attribute), “Foot structure (body structure)”*),	
	(*Associated morphology (attribute), “Lesion (morphologic abnormality)*”)}	
Injury of jugular vein (disorder)	{(*Is a, “Injury to blood vessel of neck (disorder)*”)}	injury of jugular vein/A27160412(MEDCIN)/C0347703
	{(*Is a, “Injury of systemic vein (disorder)*”)}	
	{(*Finding site (attribute), “Structure of jugular vein (body structure)*”}	
	{(*Finding site (attribute), “Structure of vein of neck (body structure)”*),	
	(*Associated morphology (attribute), “Damage (morphologic abnormality)*”)}	

The PubMed-based validation resulted in 1,265 out of 23,031 potential missing concepts being validated by biomedical literature. Table 5 demonstrates five examples of missing concepts validated through PubMed. For example, the missing concept with the FSN “*Pulmonary artery operation (procedure)*” was found to be existing in the article with the PubMed ID (PMID) 20072857. This missing concept was found in the title of this article: “Lung cysts following pulmonary artery operations: diagnostic and therapeutic implications”.

**Table 5:** Five example of missing concepts validated by biomedical literature in PubMed

Missing concept FSN	Missing concept logical definition	PMID/sentence mentioning the missing concept
Pulmonary artery operation (procedure)	{(*Is a, “Operation on the pulmonary trunk and arteries (procedure)*”)}	20072857/“Lung cysts following pulmonary artery operations: diagnostic and therapeutic implications”
	{(*Method (attribute), “Surgical action (qualifier value)”*),	
	(*Procedure site (attribute)*,	
	“*Pulmonary artery structure (body structure)*”)}	
Atrophy of choroid (disorder)	{(*Is a, “Choroidal atrophy (finding)*”)}	1205676/“... escapes from one of the viscera to lodge in the sensitized area of the atrophic choroid ...”
	{(*Is a, “Choroidal degeneration (disorder)*”)}	
	{(*Finding site (attribute), “Choroidal structure (body structure)”*),	
	(*Associated morphology, “Atrophy (morphologic abnormality)*”)}	
Left ventricular septal defect (disorder)	{(*Is a, “Left ventricular abnormality (disorder)*”)}	11083711/“... repair of a left ventricular septal defect after acute myocardial infarction.”
	“*Left cardiac ventricular structure (body structure)”*),	
	(*Associated morphology*,	
	“*Morphologically abnormal structure (morphologic abnormality)*”)}	
Peripheral arterial stent-graft (physical object)	{(*Is a, “Peripheral artery stent (physical object)*”)}	10674453/“Intravascular ultrasound evaluation of peripheral arterial stent-grafts”
	{(*Has device intended site (attribute)*,	
	“*Structure of peripheral artery (body structure)*”}	
Focal cortical cataract (disorder)	{(*Is a, “Cortical cataract (disorder)*”)}	29961989/“Focal cortical cataract due to caterpillar hair migration”
	{(*Finding site (attribute)*,	
	“*Structure of cortex of lens (body structure)”*),	
	(*Associated morphology*,	
	“*Abnormally opaque structure (morphologic abnormality)*”)}	

Regarding validation using a newer version of SNOMED CT, there were 14,329 newly added concepts in the newer September 2022 US Edition compared with the March 2021 US Edition. We found that 138 missing concepts identified by our approach exist in the newer version. Out of these, 29 missing concepts had the same FSN and the same logical definitions as with the matched concepts in the newer SNOMED CT release. In 62 out of the 138 validated missing concepts, matching concepts with the same FSNs were found in the newer SNOMED CT release, however, with different logical definitions. On the other hand, the logical definitions of 47 validated concepts were matched with the logical definitions of concepts in the newer SNOMED CT release, however, their FSNs were found to be different. Table 6 demonstrates five examples of missing concepts that were validated this way. For instance, the missing concept “*Pain of right knee region*” had the same FSN and the logical definitions as the concept with the SNOMED CT identifier 468241000124105 in the September 2022 US Edition of SNOMED CT. In addition, there are 2 separate cases where the predicted semantic tag is different from the semantic tag of the SNOMED CT concept. These were not considered to be validated by this method.

**Table 6:** Five examples of missing concepts validated by new release of SNOMED CT

Missing concept FSN	Missing concept logical definitions	Matched SNOMED CT concept ID/FSN	Matched SNOMED CT concept logical definitions
Pain of right knee region (finding)	{(*Is a, “Pain of knee region (finding)*”)}	468241000124105/Pain of right knee region (finding)	{(*Is a, “Pain of knee region (finding)*”)}
	{(*Is a, “Pain in right lower limb (finding)*”)}		{(*Is a, “Pain in right lower limb (finding)*”)}
	{(*Finding site (attribute)*,		{(*Finding site (attribute)*,
	“*Structure of right knee region (body structure)*”)}		“*Structure of right knee region (body structure)*”)}
Structure of nail unit of right thumb (body structure)	{(*Is a, “Structure of right thumb (body structure)*”)}	1162684003/Structure of nail unit of right thumb (body structure)	{(*Is a, “Structure of right thumb (body structure)*”)}
	{(*Is a, “Structure of nail unit of thumb (body structure)*”)}		{(*Is a, “Structure of nail unit of thumb (body structure)*”)}
	{(*Laterality, “Right (qualifier value)*”)}		{(*Laterality, “Right (qualifier value)*”)}
Prolapse of small intestine (disorder)	{(*Is a, “Prolapse of intestine (disorder)*”)}	1172728007/Prolapse of small intestine (disorder)	{(*Is a, “Prolapse of intestine (disorder)*”)}
	{(*Is a, “Disorder of small intestine (disorder)*”)}		{(*Is a, “Disorder of small intestine (disorder)*”)}
	{(*Finding site (attribute)*,		{(*Finding site (attribute)*,
	“*Structure of small intestine (body structure)*”),		“*Structure of small intestine (body structure)”*),
	(*Associated morphology*,		(*Associated morphology*,
	“*Prolapse (morphologic abnormality)*”)}		“*Prolapse (morphologic abnormality)*”)}
Rupture of lumbar intervertebral disc (disorder)	{(*Is a, “Lumbar disc lesion (disorder)*”)}	1145243006/Rupture of lumbar intervertebral disc (disorder)	{(*Is a, “Lumbar disc lesion (disorder)*”)}
	{(*Is a, “Lower back injury (disorder)*”)}		{(*Is a, “Injury of lumbar spine (disorder)*”)}
	{(*Is a, “Intervertebral disc rupture (disorder)*”)}		{(*Is a, “Intervertebral disc rupture (disorder)*”)}
	{(*Finding site (attribute)*,		{(*Finding site (attribute)*,
	“*Structure of lumbar intervertebral disc (body structure)”*),		“*Structure of lumbar intervertebral disc (body structure)”*),
	(*Associated morphology*,		(*Associated morphology*,
	“*Rupture (morphologic abnormality)*”)}		“*Rupture (morphologic abnormality)*”)}
Entire right half of face (body structure)	{(*Is a, “Structure of right half of face (body structure)*”)}	362626009/Entire right half of face (body structure)	{(*Is a, “Structure of right half of face (body structure)*”)}
	{(*Laterality (attribute), “Right (qualifier value)*”)}		{(*Laterality (attribute), “Right (qualifier value)*”)}

In total, 2,312 out of 23,031 (10.04%) potential missing concepts were validated by either of the three methods. Note that some potential missing concepts were validated by multiple methods. UMLS and PubMed both validated 310 potential missing concepts. UMLS and the newer SNOMED CT release both validated 5. On the other hand, 6 potential missing concepts were validated by both PubMed and the newer SNOMED CT release. All three methods together validated 3.

## Discussion

In this paper, we explored a logical definition-based approach to identify potential missing concepts in SNOMED CT. We intersected the derived logical definitions of certain candidate concept pairs to obtain the logical definitions of the potential missing concepts. A PEGASUS text summarization model was trained to predict fully specified names of the potential missing concepts. This approach has the potential to be applied to other biomedical ontologies with logical definitions.

### Significance of the approach

According to our knowledge, none of the related work in identifying missing concepts in biomedical ontologies is capable of also suggesting logical definitions of the missing concepts. Constructing logical definitions for concepts is not an easy task, requiring domain expertise as well as deep knowledge of SNOMED CT. Therefore, our approach reduces a considerable manual effort when compared with similar work. In addition, if the suggested logical definition and the corresponding concept are agreed upon by ontology curators, the concept can be automatically placed in the SNOMED CT hierarchy.

Note that even though a majority of potential missing concepts have not be validated, it does not mean that they are invalid. Such cases represent potential missing concepts without any evidence in the three sources used for validation. It should also be noted that ontology quality assurance approaches such as this are “discovery-oriented”, which means that they are supposed to uncover previously unidentified quality issues. Any quality issue identified in the process is useful to improve the ontology. Unlike many tasks involving machine learning, on our task the upper bound for metrics (e.g., accuracy, precision, recall) is not 1.0, at least not in the sense that a perfect score is the goal. Rather, since our method proposes new knowledge concepts, by necessity it will generate concepts that are not present in any of our three evaluation datasets (UMLS, PubMed, updated SNOMED CT). Instead, in future work we will look to better identify the appropriate methods for candidate concept generation that best fits with the needs of ontology curators. For now, it is clear that our method identifies a substantial number of concepts that can add value to SNOMED CT.

### Effects of normalization

As mentioned earlier, normalization was introduced to assist in matching FSNs to other FSNs/other ontology concept names/biomedical text addressing different forms the FSNs may be in. We noticed that this indeed help in identifying additional cases. For instance, when we checked whether the predicted FSNs of the potential missing concepts already existed in the same SNOMED CT release, it was seen that 4,089 FSNs matched with existing concepts. Out of the 4,089, 119 were not exact matches between the predicted FSN and an existing FSN, i.e. these were captured due to normalization. In addition, during validation, it was seen that 91 missing concepts’ names existed in the newer SNOMED CT release (29 with the same logical definitions and 62 with different logical definitions). Out of these, 14 were not exact matches.

However, it was noticed that our normalization method misses certain cases. For instance, the predicted FSN “*Closure of fistula of sclera (procedure)*” and the FSN of an existing concept “*Closure of scleral fistula (procedure)*” were normalized to “*closure fistula sclera (procedure)*” and “*closure scleral fistula (procedure)*” respectively, which are different. However, these FSNs convey the same meaning.

### Differences in the semantic tags

There are a total of 58 different semantic tags in the March 2021 US Edition of SNOMED CT. Each semantic tag corresponds to a SNOMED CT concept. For example, semantic tag “*(finding)*” has the corresponding concept “*Clinical finding (finding)*” and the semantic tag “*(procedure)*” has the corresponding concept “*Procedure (procedure)*”. In addition, there exist relations among these semantic tags. For example, semantic tag “*(finding)*” is the ancestor of semantic tag “*(disorder)*”.

While training the PEGASUS model, we noticed that in certain situations, the semantic tag predicted by the model is different from the actual semantic tag while the rest of the FSN is the same. For instance, the model predicted the FSN “*Product containing prolactin inhibiting factor (product)*” for the concept “*Product containing prolactin inhibiting factor (medicinal product)*” in the test set.

We also observed similar cases while trying to match the predicted FSNs of the potential missing concepts with the FSNs of existing concepts in SNOMED CT. For instance, as mentioned earlier, we found 169 out of 27,289 unique FSNs predicted by the model were already existing in SNOMED CT with a different semantic tag. Interestingly, in a vast majority of these cases, the semantic tag identified by our model had a relationship with the semantic tag of the existing SNOMED CT concept. In 133 cases, the predicted semantic tags were descendants of the semantic tags of the corresponding SNOMED CT concepts. For example, our model predicted the FSN “*Product containing insulin (medicinal product)*” while SNOMED CT contains a concept with the FSN “*Product containing insulin (product)*”. Here the semantic tag “(medicinal product)” is a descendant of the semantic tag “(product)”. In 24 out of 169 cases, the predicted semantic tags are the ancestors of the corresponding SNOMED CT concepts’ semantic tags. For instance, our model predicted the FSN “*Lesion of pinna (finding)*”, while SNOMED CT has a concept with the FSN “*Lesion of pinna (disorder)*”. Here, the semantic tag “(finding)” is an ancestor of the semantic tag “(disorder)”. In 34 out of 169 cases, the semantic tags do not have any relation. For instance, our model predicted the FSN “*Primary synovial chondromatosis (disorder)*”, while SNOMED CT has a concept with the FSN “*Primary synovial chondromatosis (morphologic abnormality)*”. Here, the semantic tags “(disorder)” and “(morphologic abnormality)” do not have any relation.

### Differences in the logical definitions

We also noted that in certain scenarios, when a potential missing concept’s FSN is matched to the FSN of a SNOMED CT concept, their logical definitions may not be the same. For instance, in the evaluation through the newer SNOMED CT release, we observed that 62 cases out of 91 that were validated fall into this category.

For example, Table 6 shows an example for such a case in the concept “*Rupture of lumbar intervertebral disc (disorder)*”. The difference is that the potential missing concept has the attribute-value-pair {*Is a*, “*Lower back injury (disorder)*”}, while the matched SNOMED CT concept has the attribute-value pair {*Is a*, “*Is a Injury of lumbar spine (disorder)*”}.

Out of the 91 missing concepts that existed in the newer September 2022 release of SNOMED CT, 23 of them were actually in the March 2021 version in SNOMED CT as well albeit with different FSNs and logical definitions. So, it seems that both the FSNs and the logical definitions of these concepts have been updated in the newer SNOMED CT release and the updates reflect the logical definitions and FSNs our approach suggested. For example, Table 6 shows the validated missing concept “*Entire right half of face (body structure)*” that existed in the newer September 2022 release of SNOMED CT. It was seen that this concept existed with a different FSN: “*Entire right side of face (body structure)*” and different logical definitions in the March 2021 release of SNOMED CT.

### Level distribution of concepts

**Fig. 5:**
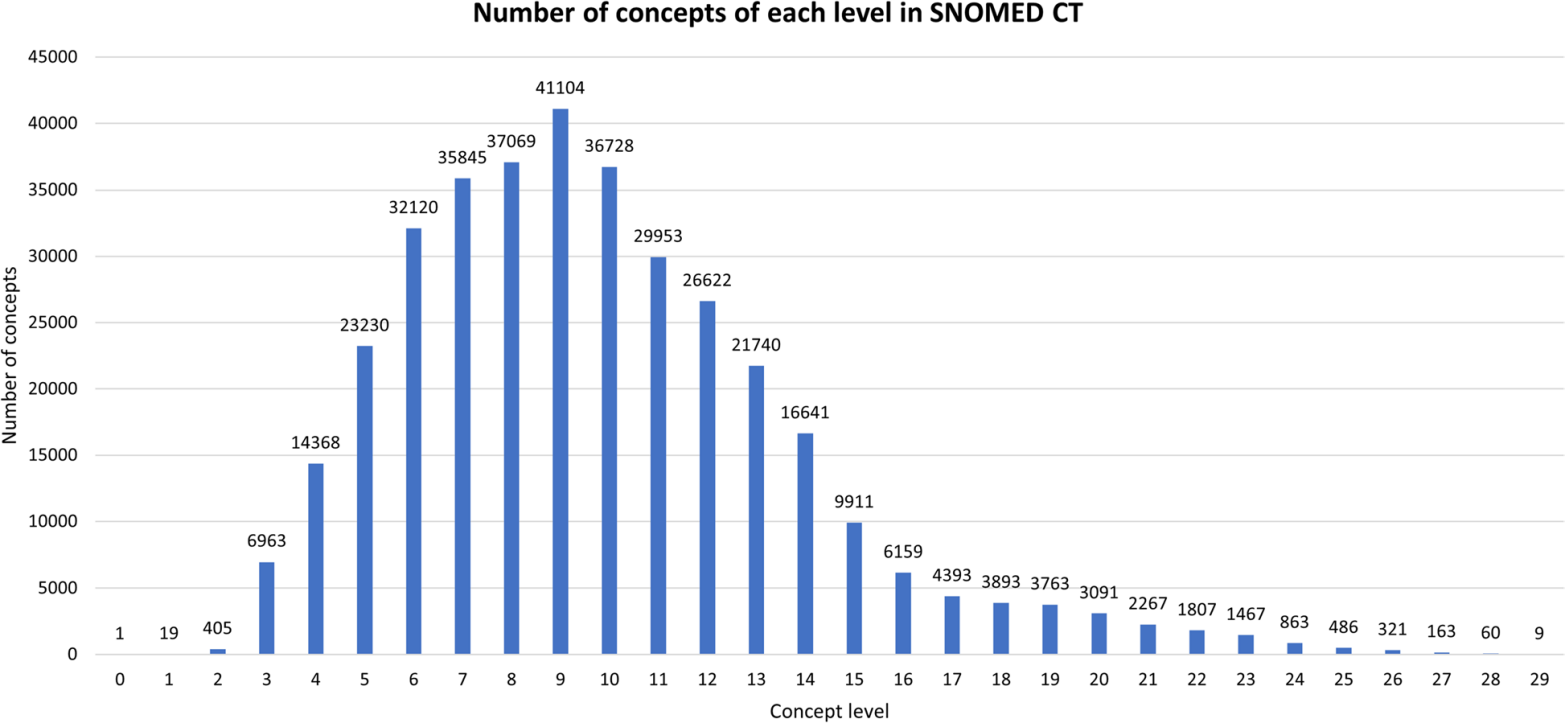
The level distribution of the SNOMED CT concepts. Out of 361,461 SNOMED CT concepts, 170,337 (47.12%) had level $\ge 10$

As mentioned previously, in this work, we considered concepts with level $\ge 10$. The reason for this was to ensure that the candidate pairs are not very general concepts. Figure 5 shows the level distribution of the SNOMED CT concepts. It was seen that out of 361,461 SNOMED CT concepts, 170,337 (47.12%) had level $\ge 10$.

### Subhierarchy distribution of potential missing concepts

We investigated the distribution of the potential missing concepts in terms of 19 subhierarchies of the SNOMED CT. Among 23,031 potential missing concepts identified, 15,820 are for the *Clinical finding* subhierarchy, 6,252 for *Procedure*, 866 for *Body structure*, 72 for *Pharmaceutical/biologic product*, 9 for *Specimen*, 5 for *Physical object*, 1 for *Situation with explicit context*, 1 for *Substance*, 1 for *Qualifier value*, and there are 4 missing concepts that the model failed to predict a semantic tag for them.

### Comparison with other summarization models

We also experimented with BART and T5 besides the PEGASUS summarization model. Table 7 demonstrates the comparison results of the three models in terms of ROUGE-1, ROUGE-2, ROUGE-L, and number of concepts correctly predicted by each model out of 36,146 concepts in the test dataset. Since PEGASUS slightly outperformed BART and T5, we leveraged PEGASUS for our downstream missing concept identification task.

**Table 7:** Performance comparison of the three models

Metric	BART	T5	PEGASUS
ROUGE-1	72.66	72.67	72.83
ROUGE-2	50.79	50.15	51.06
ROUGE-L	71.58	71.64	71.76
No. of correct prediction	11,475/36,146	11,521/36,146	11,549/36,146

### Comparison with related work

In previous work, we introduced a lexical approach based on order-preserving intersection of FSNs among candidate concept pairs in non-lattice subgraphs to identify missing concepts in SNOMED CT [43]. This previous approach identified 7,702 potential missing concepts, while the current approach discussed in this paper identified 23,031 potential missing concepts. Both approaches in common identified 1,469 potential missing concepts. For instance, the missing concept “*chronic nephritis*” was identified by both approaches. Note that this missing concept was validated by both UMLS and PubMed. However, it should also be noted that our approach in this paper was able to identify the semantic tag of this missing concept: “*(disorder)*”, which the previous approach was not capable of. In addition, the current approach also generated the logical definitions of this missing concept, which the previous approach also was incapable of.

Bodenreider has introduced an approach that leverages logical definitions and concept names to address a different quality issue in SNOMED CT: missing hierarchical relations [58]. Bodenreider’s work is also methodologically different from our work such that logical definitions are constructed from lexical features of concept names and then reasoning is performed to identify missing hierarchical relations.

Liu et al. [59] introduced a deep learning-based approach using Convolutional Neural Network (CNN) to discover missing IS-A relations in NCIt, where concept representation was generated using Doc2Vec including the concept ID, the names of its ancestors, the name of itself, the names of its children and the names of its grandchildren (if they exist). Note that logical definitions were not leveraged in Liu et al.’s work and missing IS-A relations were the focus rather than missing concepts.

### Limitations and future work

Although the fine-tuned summarization model’s ROUGE scores on the test dataset look promising (ROUGE-1/ROUGE-2/ROUGE-L: 72.83/51.06/71.76) in a text summarization context, the model only correctly predicted 11,549 out of 36,146 FSNs in the test dataset. The reason for this difference is that the ROUGE scores represent a recall measure based on n-gram overlap. In other words, the summary and the reference do not have to be the same for the ROUGE scores to be high. This is valid in text summarization tasks as there are multiple ways a summary can be written. However, since we used a normalized matching procedure to decide whether a summarized FSN is the same as the real FSN of a concept, and since our normalization is not perfect as mentioned earlier, this indicates that we could have missed a number of correct FSN predictions by the model, and the actual number of correct FSN predictions of our model could be higher than what is reported. Therefore, in the future we will explore the possibility to manually review a random sample of these cases by domain experts who are capable of identifying such cases.

In spite of the above-mentioned difference, further work is still needed to improve the summarization model’s performance for naming missing concepts based on logical definitions. In this study, we fine-tuned a pre-trained PEGASUS model to predict the FSNs from logical definitions. We will investigate whether pre-training the PEGASUS model on task specific data will have any impact on the performance. Another potential direction for improving the performance is to fine-tune the model with additional data. One fine-tuning instance could potentially be used to create multiple instances by changing the order of the input logical definitions (training data augmentation). Additionally, synonyms could also be considered during the fine-tuning process. Since, synonyms essentially have the same logical definition but different synonymous names, fine-tuning instances need to be properly adjusted to handle such cases. We would like to explore such strategies in the future.

Though 30,313 unique logical definitions were identified through derived logical definition intersection, only 23,031 unique FSNs were predicted since there were duplicate FSN predictions for different logical definitions of potential missing concepts. In such cases, we randomly removed one of the potential missing concepts with a duplicated FSN. In the future, we will investigate how to reasonably remove such duplicates as depending on their logical definitions, one may be of more importance than the other.

In this study, we chose to predict the concept’s FSN because SNOMED CT uses it to provide a unique description for the concept so that it is unambiguous, stable across multiple contexts, and optimally understandable to those whose first language is not English [10]. Identifying missing concepts’ FSNs in compliance with SNOMED CT’s concept description standard is beneficial when adding missing concepts to SNOMED CT in the future. However, since a preferred term is the most clinically appropriate way of expressing a concept in a clinical record [12], and a synonym is an acceptable way to express the meaning of a concept [11], predicting preferred terms or synonyms would also be beneficial to the comprehensiveness of SNOMED CT. For the future work, we will also investigate how to incorporate preferred terms and synonyms into our approach.

In addition, we only focused on non-lattice subgraphs of size $<10$ in this work. In the future, we would explore whether applying the same approach to all non-lattice subgraphs would be as effective.

As mentioned earlier, the fine-tuned PEGASUS model predicts different semantic tags for some of the concepts. An interesting future direction is to see whether this could be leveraged to audit the semantic tags of concepts.

Another limitation of our work is that no manual evaluation was performed to assess the actual effectiveness of the approach. We plan to submit a random sample of the identified missing concepts to SNOMED International for auditors’ manual review and potential incorporation in a new version of SNOMED CT.

## Conclusions

In this paper, we introduced an approach to identifying potential missing concepts in SNOMED CT by intersecting the derived logical definitions of unrelated concepts in non-lattice subgraphs and fine-tuning a text summarization model to predict the fully specified names of the potential missing concepts based on their logical definitions. Applied to the March 2021 US Edition of SNOMED CT, our approach identified 23,031 potential missing concepts with both fully specified names and logical definitions. Out of these, a total of 2,312 were validated through either UMLS, PubMed, or a newer release of SNOMED CT. The results indicated that our approach is encouraging and has the potential to be applied to other biomedical ontologies, even though further work is still needed to improve the model for concept naming based on logical definitions.

## Data Availability

The results for identified missing concepts are available at https://github.com/XubingHao/BMC2022_Missingconcepts.

## Abbreviations

EHR: Electronic health record
QA: Quality assurance
SNOMED CT: Systematized Nomenclature of Medicine - Clinical Terms
FSN: Fully Specified Name
MASS: Masked Sequence to Sequence pre-training
GSG: Gap sentence generation
C4: Colossal and Cleaned version of Common Crawl
FCA: Formal concept analysis
UMLS: Unified Medical Language System
DL: Description Logics
ROUGE: Recall-Oriented Understudy for Gisting Evaluation
GO: Gene Ontology
MeSH: Medical Subject Headings
HPO: Human Phenotype Ontology
